# Risk factors for dermatitis in submariners during a submerged patrol: an observational cohort study

**DOI:** 10.1136/bmjopen-2015-010975

**Published:** 2016-06-02

**Authors:** Amy Flaxman, Elizabeth Allen, Claudia Lindemann, Yuko Yamaguchi, Matthew K O'Shea, Joanne L Fallowfield, Michael Lindsay, Frances Gunner, Kyle Knox, David H Wyllie

**Affiliations:** 1 Jenner Institute, Centre for Cellular & Molecular Physiology, University of Oxford, Oxford, UK; 2 Department of Academic Medicine, Royal Centre for Defence Medicine, Birmingham, UK; 3 Environmental Medicine and Science Division, Institute of Naval Medicine, Hampshire, UK; 4 Nuffield Department of Primary Care Health Sciences, New Radcliffe House, Oxford, UK; 5 Nuffield Department of Medicine, Department of Microbiology, John Radcliffe Hospital, Oxford, UK

**Keywords:** S. aureus, Dermatitis, Submariners

## Abstract

**Objective:**

The aim of this pilot study was to determine risk factors, including *Staphylococcus aureus* nasal carriage, for dermatitis in submariners during a submarine patrol.

**Participants and methods:**

36 submariners undertaking a submerged 6-week patrol participated in the study. Severity of dermatitis and its impact was assessed using visual analogue scales and questionnaires at baseline and weekly throughout the patrol. *S. aureus* carriage levels in submariners were determined by nasal swabbing at baseline and shortly before disembarking the submarine. Occurrence of any skin or soft tissue infections (SSTI) were reported to the medical officer and swabs of the area were taken for subsequent analysis.

**Results:**

*S. aureus* carriers were significantly more likely than non-carriers to have previously received treatment for a cutaneous abscess (39% vs 5%, OR=13 (95% CI 1.3 to 130)) with a trend to being submariners longer (p=0.051). Skin scores at baseline and on patrol were not significantly associated with carriage status. Higher dermatitis scores were observed in those who had been submariners longer (p=0.045). Smoking and allergies were not found to be linked to carriage status or skin health score in this cohort.

**Conclusions:**

This small pilot study investigates *S. aureus* carriage status and skin health in submariners. Length of submarine service but not *S. aureus* carriage was identified as a risk factor for worsening skin health in this small cohort during a 6-week patrol. This does not support *S. aureus* decolonisation to improve skin health in this population. Further investigation into causes of dermatitis in submariners is required. This data supports a better understanding of the potential impact of exposure to environmental factors that could affect skin health in submariners.

**Strengths and limitations of this study**

- This study investigates skin health and *Staphylococcus aureus* carriage in submariners, in whom incidence of poor skin health and infection due to *S. aureus* has been previously reported.
- The submarine patrol observed in this study was relatively short, at 40 days, which may impact results seen and conclusions drawn.
- This a small pilot study and does not have a civilian control cohort which would be useful for comparison purposes.
- We have introduced a new visual analogue scale for quantitative, longitudinal assessment of skin health, which could be used in further studies and other occupational health settings.

## Introduction

Observational studies on UK and US submariners have noted that skin disorders, including infections, are common during submarine patrols.^1^ ^2^ In a retrospective review of UK Vanguard Class submariners, dermatological disorders were among the most common causes of medical consultation with a prevalence of 10–20% during a patrol.^3^ Rates of skin infections due to *Staphylococcus aureus* (*S. aureus)* were far higher than those observed in a civilian population of similar age.^4^

*S. aureus* is a Gram-positive bacterium colonising the nares of about a third of the general population.^5^ While nasal colonisation often has no apparent clinical manifestations, risk of *S. aureus* disease is increased in carriers relative to non-carriers.^6^ In particular, skin and soft tissue infections (SSTI) are commonly caused by *S. aureus*; for example, in one population, 80% of individuals with skin lesions were found to be *S. aureus* nasal carriers, with 65% having the same phage type in the nose lesions.^7^ Similarly, atopic eczema is more common in *S. aureus* carriers^8^ and appears to be driven by *S.* *aureus*-derived peptides.^9^

Multiple *S. aureus* strains exist, and particularly virulent strains can be acquired under environments where there is close population contact, such as households^10^ and military facilities. Transmission probably occurs through either skin-to-skin contact or fomites, such as shared clothes and /towels.^11^ In a submarine, aerosolised transmission is also possible.^12^ Despite the high frequency of skin disorders in the submarine environment, few studies linking this to *S. aureus* carriage have been carried out; a study performed in the early 1970s indicated that, similar to civilian populations, *S. aureus* carriage rates in submariners are about 30%.^12^

Evidence from randomised trials supports the use of decolonisation for the prevention of *S. aureus* infection in selected civilian and military populations at high risk of *S. aureus* disease.^13–16^ It is unclear, however, what proportion of the skin disorders of submariners are associated with *S. aureus* carriage or infection, as opposed to being due to other aspects of the unusual environment in the submarine, such as the use of cleaning chemicals.

Since prospective epidemiological studies investigating risk factors for dermatological disorders have not been carried out in this population, we performed a prospective cohort study monitoring the skin health, and investigating its determinants, during a submarine patrol.

## Materials and methods

### Study participants and study design

Samples used in this study were collected during a prospective cohort study in UK submariners, undertaken as part of the Royal Navy's occupational health programme. A total of 50 serving Royal Navy submariners provided written consent, and a nasal swab was taken between 24 and 72 hours later. Samples were collected from submariners who had a period of on-shore training and vacation, prior to deployment. A second nasal swab was obtained from submariners, by the medical officer on board, before disembarking.

### Assessment of skin health

Assessment of skin health was carried out using two methods. First, participants used a visual analogue score to score two aspects of their skin health: ‘skin generally’ and ‘hands’ skin. Second, participants used a validated instrument to score the severity of any dermatitis on their hands as clear, almost clear, moderate, severe or very severe.^17^

### Skin health data collection

At least 24 hours after recruitment to the study, submariners completed a prepatrol skin health questionnaire, which included a baseline skin health diary. While on patrol, submariners completed a weekly skin health diary (see online supplementary information). If any skin or soft tissue infections (SSTI) occurred, they reported to the medical officer and the area was swabbed using charcoal swabs (Technical Service Consultants Ltd, TS/5-18) and stored at 4°C for subsequent analysis.

10.1136/bmjopen-2015-010975.supp1 Supplementary data

### Determining *S. aureus* carriage densities using nasal swabs

Nasal swabbing was performed as previously described^18^ using viscose breakpoint swabs (Technical Service Consultants Ltd, TS/19-S). Each swab was then snapped off into 2 mL sterile PBS (Sigma-Aldrich, D8862) in bijoux, which were stored and transported at 4°C. All swabs were processed within 4 days and 10 hours of being taken. Each bijoux was vortexed, and 50 µL of sample was spirally plated onto horse blood agar (Oxoid Ltd, PB0114A) and Brilliance Staph 24 (Oxoid Ltd, PO1186A) agar plates using Autoplate Automated Spiral Plater (Advanced Instruments, Inc). Plates were read using QCount Automated Colony Counter (Advanced Instruments, Inc) after 24 and 48 hours. A total of 400 µL of sample from each bijoux was also enriched for 24 hours at 37°C in 5% salt broth (Oxoid Ltd, EB1296E) before being plated onto Brilliance Staph 24 agar plates as described above. Suspected positive *S. aureus* colonies on Brilliance Staph 24 plates were confirmed using Staphylase Test Kit (Oxoid Ltd, DR0595). All data show bacterial recovery in cfu/mL. We optimised the protocol by spiking *S. aureus* into PBS, storing for up to 1 week at 4°C and then processing as described above. Colony counts remained stable under these conditions. To investigate whether the swabs had been inoculated correctly, the total aerobic counts were measured by plating the material on blood agar.

### Statistical analysis

Bacterial count data were log transformed and analysed using paired t-tests. Mann-Whitney tests were used to compare continuous variables. For dichotomous variables, when comparing two factors, results were expressed as ORs with 95% CIs. Logistic regression was used to compare dichotomous and continuous outcomes. Categories of skin scores (ie, mild, moderate, severe) were compared with continuous variables using nominal regression (ie, the probabilities of these categories were considered separately, rather than as a scale). Missing data and non-response rates are shown throughout.

IBM SPSS Statistics V.22 was used for exploratory data analysis and descriptive statistics of bacterial count data and for all statistical analyses above.

GraphPad Prism software V.6.03 (GraphPad Software, Inc) was used for graphical representation of bacterial count data and skin score data. R V.3.1.1 for Windows was used for graphical representation of skin scores from individual submariners.

### Ethical statement

The study was approved by the UK Ministry of Defence Research Ethics Committee (MODREC) protocol reference number 0903/228.

## Results

### Recruitment

**Figure 1:**
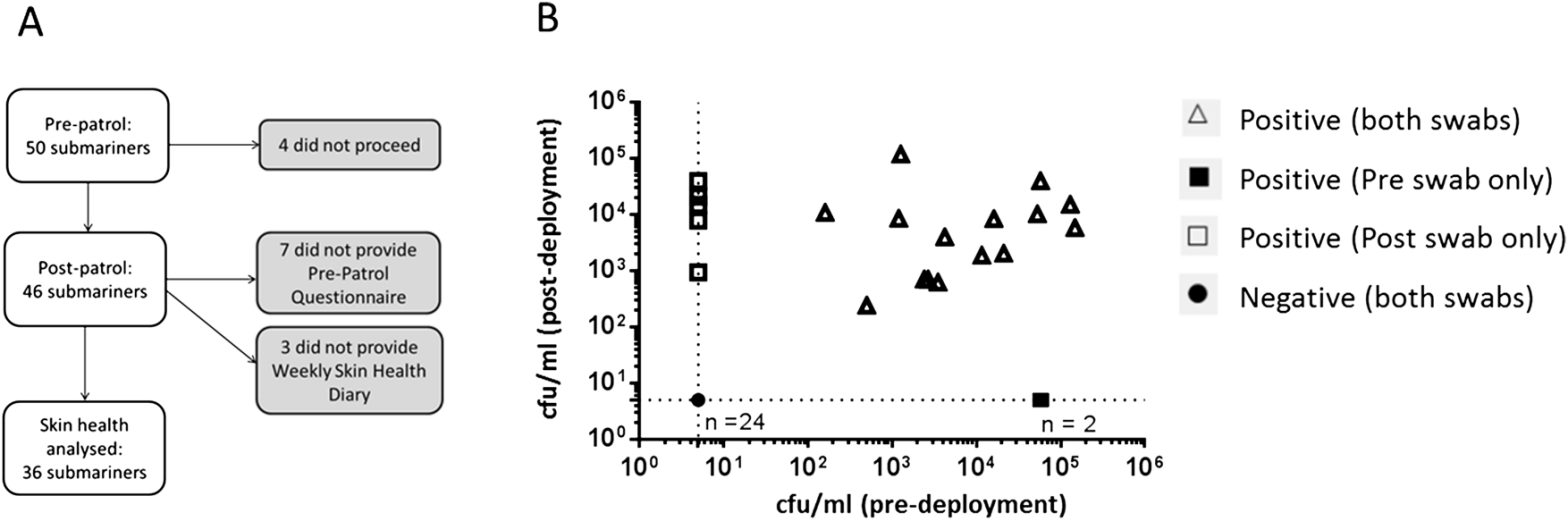
(A) Flow chart showing study design. (B) *S. aureus* carriage status of submariners. *S. aureus* recovery from predeployment (x-axis) and postdeployment (y-axis) nasal swabs (cfu/mL). Negatives are plotted at detection limit shown by dotted lines.

A total of 50 submariners on a Royal Navy Trafalgar Class submarine were recruited to participate in the study in the period just before the submarine began a patrol. All participants were eligible for inclusion. There were no exclusion criteria. Questionnaires were administered and nasal swabs were obtained in the 48 hours preceding the start of the patrol. The nasal swabs were transferred to a research facility and analysed as a batch. A total of 46 of the original 50 submariners provided nasal swabs before disembarking from the submarine at the end of the patrol; 40 days after the patrol began (figure 1A).

### Quality control

The colony counts for the 50 baseline swabs and 46 postpatrol swabs were 3.86±0.83 log_10_ cfu/mL and 3.99±0.49 log_10_ cfu/mL (mean and SD), respectively. The duration of sample storage as calculated by spike recovery experiments (see Methods) prior to plating was not associated with total aerobic counts (Kruskal-Wallis test comparing cfu/mL from swabs taken on days 2, 1 and 0 relative to submarine departure, p=0.183). Therefore, we concluded that the samples had been taken correctly.

### *S. aureus* in submariners

In total, 24/46 (52.2%) submariners were negative for *S. aureus* predeployment and postdeployment, 2/46 (4.3%) submariners were positive for *S. aureus* predeployment only and 5/46 (10.9%) submariners were positive for *S. aureus* postdeployment only (figure 1B). In total, 15/46 (32.6%) were positive for *S. aureus* predeployment and postdeployment. Mean *S. aureus* burden (assessed by quantitative nasal counts, considering negative samples to be at a limit of detection of 5 cfu/mL) was 1.90 log_10_ cfu/mL prepatrol and 2.02 log_10_ cfu/mL postpatrol (difference=−0.120, 95% CI −0.569 to 0.329). No increase in *S. aureus* bacterial recovery was seen after the patrol (p=0.593).

Submariners were then categorised by their *S. aureus* carriage status based on results of previous studies, which indicated that nasal carriers and non-carriers were discrete entities, based on immunological analyses.^19^ In particular, individuals with intermittent carriage and those without detected carriage appeared similar to each other, but discrete from persistent carriers.^19^ Therefore, in this analysis, those with two positive swabs were ‘persistent carriers’; and those with either one or no *S. aureus* positive swabs were termed ‘non-carriers’.

### Skin health assessment at baseline

Complete skin health data and *S. aureus* carriage status data were available for 36 individuals (skin health data from 14 individuals was either unavailable or unusable due to incomplete data sets) (figure 1A). Demographic and skin health data from these 36 individuals were analysed by *S. aureus* carriage status (table 1). Carriers were significantly more likely to have previously had a cutaneous abscess incised and drained compared with non-carriers (39% vs 5%, OR=13 (95% CI 1.3 to 130). Increased age, time spent in the Royal Navy and time as a submariner all trend towards an association with *S. aureus* carriage. Smoking, vaccination status, allergies, recent infection and naval rank were not significantly associated with carriage status in this study.

**Table 1:** Characteristics of submariners recorded in prepatrol skin health questionnaires stratified by *S. aureus* nasal carriage and skin health scores

Demographics (ratio)	*S. aureus* carriage status			Skin health score			Non-response rate
	Carriers (n=13)	Non-carriers (n=23)	p Value/OR and 95% CI	High (n=17)	Low (n=19)	p Value/OR and 95% CI	
Male	1.000	1.000	1.000	1.000	1.000	1.000	0.000
Median age (years)	34.000	28.000	0.073	35.000	28.000	0.041	0.028
Median time in navy (years)	14.000	5.000	0.051	13.000	6.000	0.042	0.000
Median time as submariner (years)	14.000	3.000	0.087	12.000	3.000	0.045	0.000
Officer	0.231	0.217	0.926 (0.182 to 4.711)	0.294	0.158	0.450 (0.089 to 2.263)	0.000
Marine engineer	0.231	0.304	1.458 (0.305 to 6.984)	0.294	0.263	0.857 (0.199 to 3.690)	0.000
Current smokers	0.308	0.304	1.016 (0.232 to 4.441)	0.471	0.158	4.471 (0.998 to 22.517)	0.000
Asthma	0.083	0.091	0.909 (0.074 to 11.194)	0.118	0.059	2.133 (0.175 to 26.033)	0.056
Eczema	0.000	0.045	n/a	0.000	0.059	n/a	0.056
Hayfever	0.083	0.174	0.432 (0.043 to 4.366)	0.118	0.167	0.667 (0.097 to 4.579)	0.028
Surgery in past month	0.000	0.043	n/a	0.059	0.000	n/a	0.028
Vaccination in past month	0.000	0.000	n/a	0.000	0.000	n/a	0.028
Flu-like illness in past month	0.000	0.136	n/a	0.125	0.053	2.571 (0.211 to 31.326	0.028
Cutaneous abscess on skin in past month	0.154	0.048	3.636 (0.295 to 44.775)	0.067	0.105	0.607 (0.050 to 7.415)	0.056
Skin infection in past month	0.000	0.000	n/a	0.000	0.000	n/a	0.083
Ever had a cutaneous abscess incised and drained	0.385	0.045	13.125 (1.321 to 130.424)	0.313	0.053	8.182 (0.842 to 79.535)	0.028
Antibiotic use in past 6 months	0.154	0.000	n/a	0.125	0.000	n/a	0.028
Received all childhood vaccines	1.000	0.905	n/a	1.000	0.889	n/a	0.083
Ever had antibiotics post surgery	0.385	0.182	2.813 (0.593 to 13.336)	0.250	0.263	0.933 (0.203 to 4.285)	0.028
Previous *S. aureus* infection	0.000	0.000	n/a	0.000	0.000	n/a	0.083
Deployed within last 3 months	0.923	0.909	0.833 (0.068 to 10.202)	0.875	0.947	2.571 (0.211 to 31.326)	0.028
Chlorhexidine allergy	0.000	0.000	n/a	0.000	0.000	n/a	0.028
Persistent *S. aureus* carrier	n/a	n/a	n/a	0.471	0.263	2.489 (0.616 to 10.056)	0.000

Mann-Whitney tests shown for continuous variables and OR with 95% CIs shown for dichotomous variables.

### Skin health assessment during the patrol

**Figure 2:**
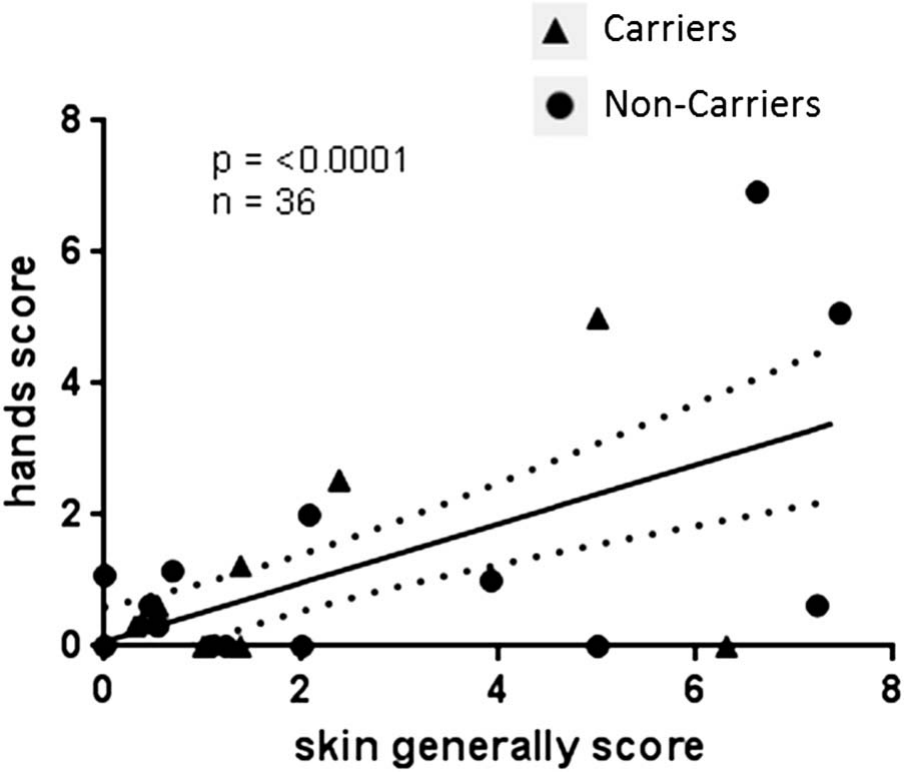
Baseline skin health submariners scored their general skin health (x-axis) and hands skin health (y-axis) on a scale of 0–10 before the patrol. Linear regression with 95% CIs shown.

**Figure 3:**
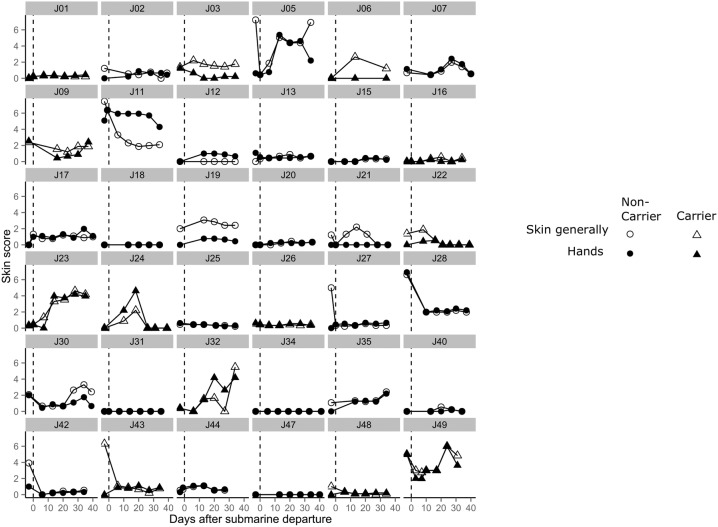
Skin health during patrol general skin health and hands skin health scores (on a scale of 0–10) for each submariner (with carriage status shown) from baseline and throughout course of patrol.

We analysed self-assessed skin health scores for 36 submariners at baseline and throughout the patrol. Figure 2 shows that self-assessed skin scores at baseline for the categories ‘skin generally’ and ‘hands’ show positive correlation (r^2^=0.41, p<0.0001), suggesting that factors relevant to the whole skin, rather than just the hands, may be important. Figure 3 shows the self-assessed skin scores for each submariner predeployment and throughout the course of the patrol, according to *S. aureus* carriage. Several different patterns were suggested by this pilot study. Some individuals, such as J05, J23 and J49, reported worsening skin scores over time on patrol. J1, J25 and J47 reported very low skin scores at baseline with no changes in skin scores over time on patrol. J09 and J19 reported slightly higher baseline skin scores, which did not worsen over the course of the patrol. Some individuals reported higher scores at baseline, with consistently lower scores throughout the course of the patrol (J27, J28 and J43). J11was the only submariner for which skin health improved over the course of the patrol.

We determined whether hand skin appearing as ‘normal’ or ‘not normal’ changed during the submerged patrol using logistic regression. When all individuals were modelled together, there was no significant change in hand skin appearance during the patrol (p=0.283). However, since different individuals may respond differently over time, models were fitted for each individual. Analysed in this way, J5, J11, J23 and J35 showed significant differences in hand skin appearance during the patrol, with J5, J23 and J35 worsening and J11 improving. We used nominal logistic regression to analyse dermatitis severity over time using the scores from a picture scale. Compared with submariners scoring ‘Clear’, there was a significant increase in ‘Moderate’ but no significant increase in ‘Almost Clear’ during the course of the patrol (p=0.029). Fitting models for data from each individual, J17 and J23 showed significantly increased ‘Almost Clear’ scores and J35 and J49 showed significantly increased ‘Moderate’ scores. Reassuringly, these results were concordant with those seen for these individuals when analysing skin health using the visual analogue scale (figure 3).

**Figure 4:**
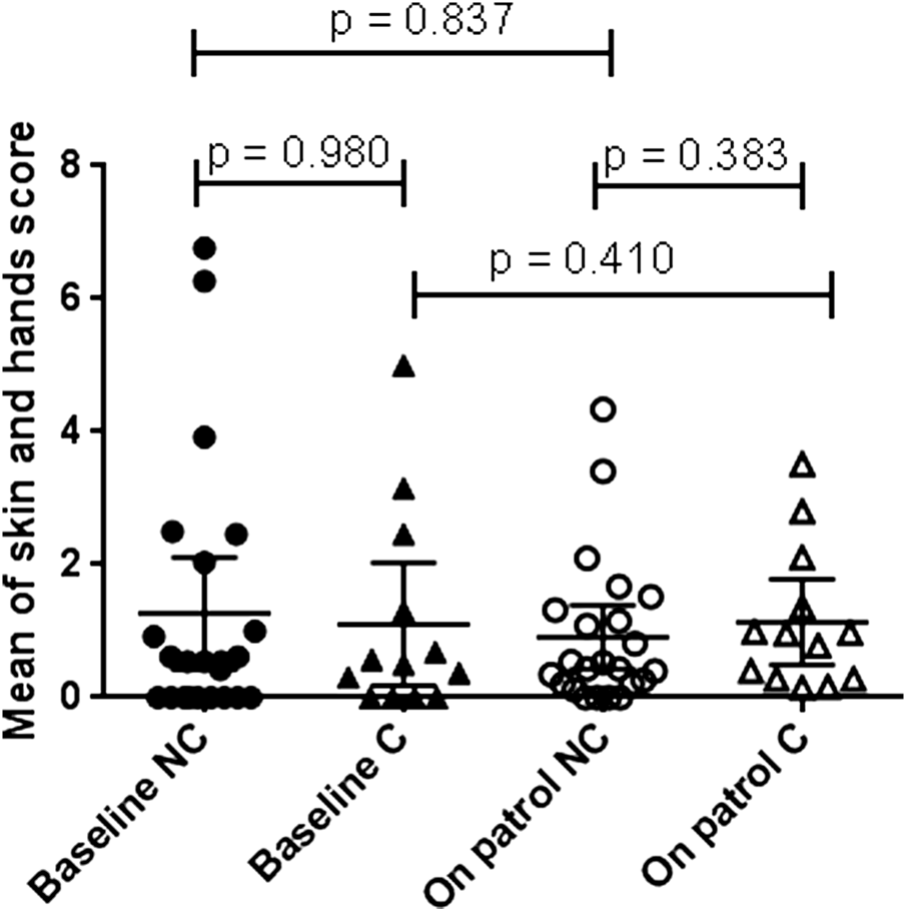
Relationship between skin health and *S. aureus* carriage status. Mean score of general skin health and hands skin health at baseline was calculated and is shown for non-carriers (NC, filled circles) and carriers (C, filled triangles). Mean score of general skin health and hands skin health at all time points post 0 was calculated and is shown for NC (empty circles) and C (empty triangles). Mean and 95% CI shown. p Values calculated using Mann-Whitney test.

Since ‘skin generally’ and ‘hands’ skin scores follow the same patterns in most submariners, we averaged the ‘skin generally’ and ‘hands’ skin scores. We used this as a summary measure of skin health at baseline, and also at each time point during the patrol. ‘On patrol’ and baseline skin health scores were positively correlated (data not shown). We investigated whether carrier status impacted on skin health score (figure 4). We found no difference between skin scores in carriers and non-carriers at baseline (p=0.980) nor ‘on patrol’ (p=0.383). We also found no change in skin scores from baseline to ‘on patrol’ of carriers (p=0.410) and non-carriers (p=0.837).

In an exploratory analysis, we further assessed skin scores by dichotomising the averaged ‘on patrol’ scores into high (>0.7) and low (<0.7), a cut-off chosen to divide the group approximately in half. Seventeen submariners were classed as ‘high’ (ie, having worse skin on patrol) and 19 were classed at ‘low’ (ie, having few skin problems while on patrol). We then assessed various demographic measures related to skin health in relation to high or low skin health score (table 1). Submariners with high skin scores were older (p=0.041), had been in the Royal Navy for longer (p=0.042), had been submariners for longer (p=0.045 and had a trend towards being more likely to have a cutaneous abscess incised and drained (31% vs 5%, OR=8.0 (95% CI 0.74 to 80)). Smoking, vaccination status, recent infection, allergies and rank were not significantly associated with high or low skin score in this study.

Only one submariner, J09, reported an SSTI infection on his foot. Swabs were taken on day 18 of the patrol, but were unable to recover *S. aureus* from these swabs either by direct plating or after enrichment.

## Discussion

We studied *S. aureus* nasal carriage in a unique cohort of submariners during a 6-week patrol at sea. A total of 33% of submariners were *S. aureus* nasal carriers at baseline and at the end of the patrol. This is in line with *S. aureus* nasal carriage studies within civilian community populations and UK military Royal Marine recruits.^20^ ^21^ When we analysed *S. aureus* carriage by baseline variables, we found that carriers were significantly more likely to have had a cutaneous abscess incised and drained. *S. aureus* nasal carriage is reported as the primary risk factor in recurrent furunculosis,^22^ and while the causative micro-organisms in past abscesses of submariners are not known, *S. aureus* carrier status is likely to have been a factor in causing abscesses. We found no indication that *S. aureus* nasal carriage was associated with increased severity of skin health either at baseline or while on patrol as determined by our self-assessed scoring system.

We identified three factors associated with poor skin health in submariners. Higher skin health scores were positively correlated with increasing age, time in the Royal Navy and time as submariner. However, age, duration of Royal Navy service and time spent as submariner all positively correlated in this cohort, and therefore, it was not clear whether age alone or time spent as a submariner may be risk factors for *S. aureus* nasal carriage and worsening skin health while on patrol. Since we do not have a control population, who were not repeatedly exposed to the submarine's environment, we cannot exclude the possibility that skin health worsens with age. However, an alternative possibility is that repeated exposure to compounds present in the environment of the submarine causes sensitisation or irritation in some individuals, leading to an allergic or irritant dermatitis. If so, these compounds may be causing widespread (rather than just hand) dermatitis, as judged by the strong correlation between ‘skin generally’ and ‘hands’ skin scores, and may be from a source to which the whole crew was exposed, since we did not find a significant association between occupational role while at sea and worsening skin health.

### Limitations

Our study is relatively small. Larger studies with a relevant control group could be used to further test the ‘occupational allergen’ hypothesis outlined above, as could skin patch testing if candidate allergens could be proposed. It could also be used to determine whether the different patterns of skin health we documented (figure 3) are observed in other settings.

We were unable to assess SSTI risk factors using analytical methods as only one infection occurring. We would have expected *S. aureus* infection to be a contributor to SSTI morbidity, as has been observed previously during submarine patrols, but the short duration of the study may have limited number of SSTIs.

### Future research

Future studies could consider modifications to enhance the performance of our skin health assessment tool, including taking more than one baseline measurement and familiarisation with the instrument during the patrol, which were not feasible in this study for operational reasons. Such steps might increase the consistency of questionnaire scoring. Additionally, validation of the visual analogue scale by an independent expert (such as the medical officer) in a cohort could be considered, although our visual analogue scale method appeared to perform well relative to a previously validated picture-based method of dermatitis assessment,^17^ which was used in parallel in the present study.

This study could provide a basis for future studies with similar goals in other environments where exposure to allergens and/or SSTIs were common, such as military training centres and other closed communities.^23^

## Conclusions

We used two instruments to measure self-reported skin health in a cohort of submariners. Our instrument and one that had been previously validated correlated well with each other. A significant association between worse skin health, age and duration as a submariner was detected. One explanation for the patterns of skin health reported in this study could involve sensitisation or irritation of a proportion of submariners to an environmental compound. *S. aureus* carriage was not associated with dermatitis in this cohort, a result which does not support *S. aureus* decolonisation as a method of improving skin health in this population. The tools described here to measure *S. aureus* carriage status and skin health in submariners were effective and could be used in future studies.

### Supplementary information

Templates of prepatrol skin health questionnaire and Weekly Skin Health Diary.
