# Supplementary material for: Risk factors for dermatitis in submariners during a submerged patrol: an observational cohort study
Source: BMJ Open. 2016 Jun 2;6(6):e010975. doi: 10.1136/bmjopen-2015-010975 (PMC4893864; doi:10.1136/bmjopen-2015-010975)
Supplement: Supplementary data [file bmjopen-2015-010975supp.pdf]

Pre-Patrol Skin Health Questionnaire

Name: ______________________________

Date: ______________________________

Submarine: ______________________________

1. Date of baseline swab: **Day** **Month** **Year**

1. Date of Birth / /

2. Sex Male ☐ Female ☐

3. How long have you been in the Royal Navy? ____________________

4. How long have you been a submariner? ____________________

5. What is your rank or rate? ____________________

6. What is your branch and/or specialty? ____________________

7. While alongside, do you smoke?

Yes		
	If so, number per day	
No		

8. Do you have any allergies? If so, to what?

9. Did you have any of the following conditions diagnosed before joining the Royal Navy?

Condition	**Yes/No**	**If yes, for how long?**	**Do you require treatment and if so what medication(s) and how often?**
**Asthma**			
**Eczema (Dermatitis)**			
**Hayfever**			

10. Have you undergone either of the following in the past month?

Procedure	**Yes/No**	
**Surgery**		*If Yes, complete Appendix A*
**Vaccination**		*If Yes, complete Appendix B*

Continued Overleaf /

11. Have you suffered either of the following in the past month?

Procedure	**Yes/No**	**Date**
**'Flu like illness**		
**Boils or spots on the skin**		
**Other kinds of skin infection**		

12. Have you ever developed a spot or boil on the skin, which you or other people had to squeeze, open with a needle or cut open?

Yes ☐ No ☐ Don't know ☐

If yes: approximately how long ago? ______________________

13. Have you ever received any antibiotics by mouth in the past 6 months?

Yes ☐ No ☐ Can't remember ☐

14. If yes: Please give name of last antimicrobial: ______________________

Please give approximate date of last antimicrobial prescription: / /

15. Did you receive the recommended childhood vaccines?

Yes ☐ No ☐ Can't remember ☐

16. Have you ever had to take antibiotics to control an infection after surgery?

Yes ☐ No ☐ Don't know ☐

If yes: approximately how long ago? ______________________

17. Do you know whether you have had an infection due to *Staphylococcus aureus* previously?

Yes ☐ No ☐ Don't know ☐

If yes: approximately how long ago? ______________________

18. Have you deployed on a submarine before this planned patrol in the last

Week Yes ☐ No ☐ Don't know ☐
Month Yes ☐ No ☐ Don't know ☐
3 months Yes ☐ No ☐ Don't know ☐

19. Have you ever been diagnosed with an allergy to chlorhexidine (a chemical used for skin cleaning)

Yes ☐ No ☐ Don't know ☐

Continued /

20. Now, please complete the below skin condition diary entry. You will be asked to fill this in weekly during the patrol.

BASELINE SKIN HEALTH QUESTIONNAIRE

In the last week, did you…

	Answer	Example answer
have pain, irritation or discomfort in your hands or other skin areas ?		Yes or no
have worse pain, irritation or discomfort in your hands or other skin while doing your work?		Yes or no
have pain, irritation or discomfort in your hands or other skin which prevented you doing your work?		Yes or no
have to see the medical team (MO, MA) about the skin problem?		Yes or no
Put creams or steroids on your skin?		Yes or no
Have to take any kind of antibiotic?		Yes or no
Have to take any kind of antibiotic for your skin?		Yes or no

On a typical day in the last week,

	Answer	Example answer
How many showers did you have per day?		A number
Did you use a towel used by more than one person		Yes, or no
Did you clean a compartment or equipment with cleaning chemicals?		Yes, or no
If so, did you always wear gloves when handling these chemicals?		Yes, or no

Today:

	Answer	Example answer
How bad is your skin generally today?	Normal Very bad<br>0 ________________________________ 10	Please put a X on the line
How bad is the skin of your hands today?	Normal Very bad<br>0 ________________________________ 10	Please put a X on the line
Is your hand skin normal today?		Yes or no

Continued Overleaf /

If no, please look at the pictures below. Which picture does the worst affected hand most resemble?		Clear,<br>Almost clear,<br>Moderate,<br>Severe,<br>Very severe

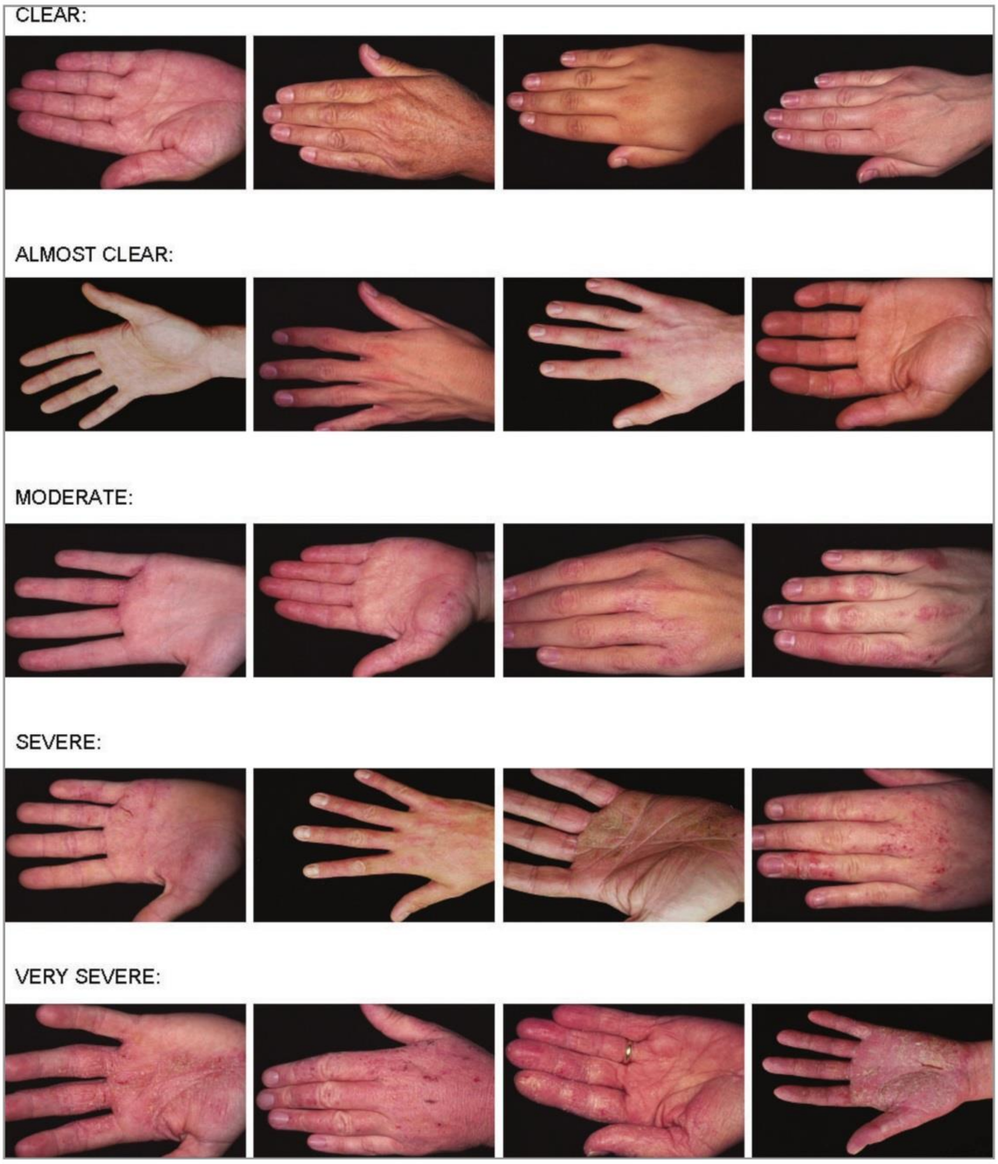

End

Appendix A – Details of surgery in past month

Date of surgery	*Surgical procedure*

Appendix B – Details of vaccination in past month

Date of vaccination	*Vaccine name*

Weekly Skin Health Diary.

Name: ______________________________

Date: ______________________________

Submarine: ______________________________

Week of Deployment: , Ending (Date): ____________________

	**Answer**	**Example answer**
have pain, irritation or discomfort in your hands or other skin areas ?		Yes or no
have worse pain, irritation or discomfort in your hands or other skin while doing your work?		Yes or no
have pain, irritation or discomfort in your hands or other skin which prevented you doing your work?		Yes or no
have to see the medical team (MO, MA) about the skin problem?		Yes or no
Put creams or steroids on your skin?		Yes or no
Have to take any kind of antibiotic?		Yes or no
Have to take any kind of antibiotic for your skin?		Yes or no

On a typical day in the last week,

	Answer	Example answer
How many showers did you have per day?		A number
Did you use a towel used by more than one person		Yes, or no
Did you clean a compartment or equipment with cleaning chemicals?		Yes, or no
If so, did you always wear gloves when handling these chemicals?		Yes, or no

Continued Overleaf /

Today:

	Answer	Example answer
How bad is your skin generally today?	Normal Very bad<br>0 ________________________________ 10	Please put a X on the line
How bad is the skin of your hands today?	Normal Very bad<br>0 ________________________________ 10	Please put a X on the line
Is your hand skin normal today?		Yes or no

If no, please look at the pictures below. Which picture does the worst affected hand most resemble?		Clear,<br>Almost clear,<br>Moderate,<br>Severe,<br>Very severe

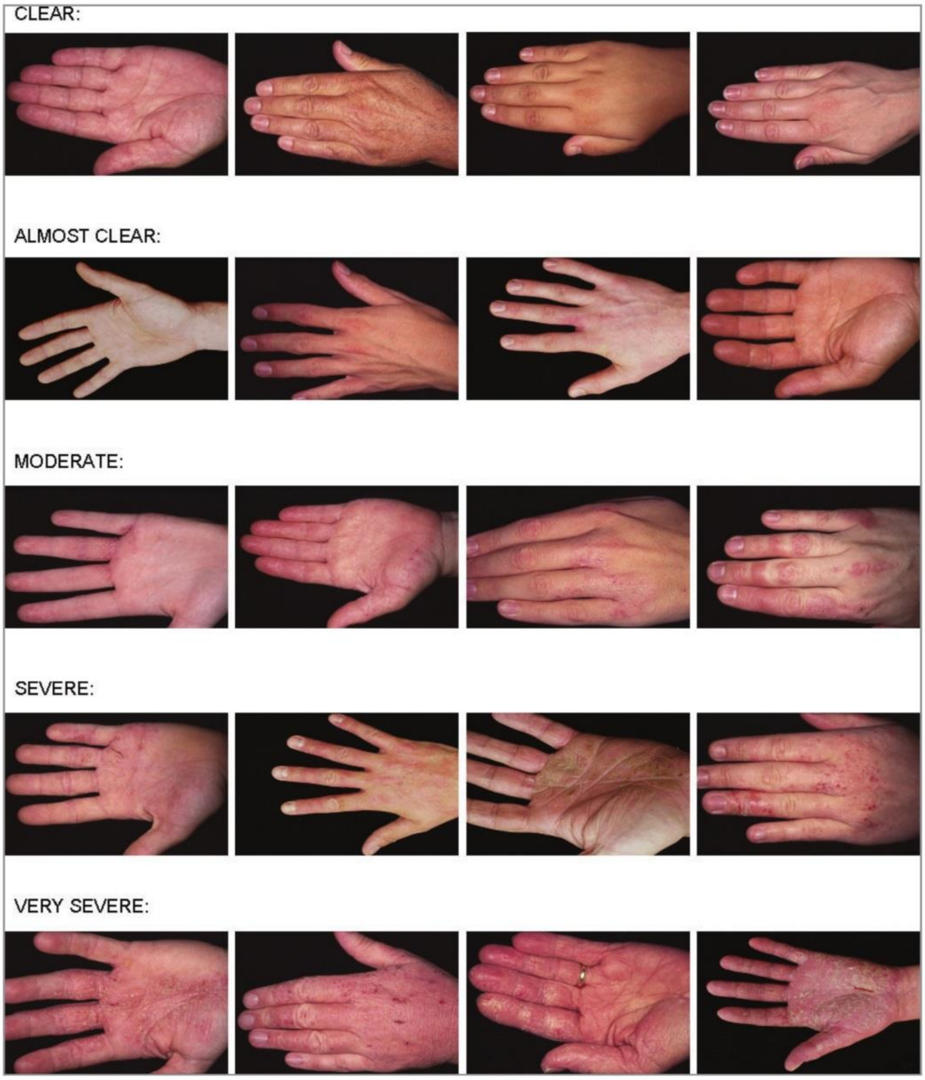

End
